# Chemical and Enantioselective Characterization and Preliminary Biological Activities of *Aristolochia lingulata* Aerial-Part Essential Oil

**DOI:** 10.3390/plants15172692

**Published:** 2026-09-02

**Authors:** Diego R. Vinueza, Linda M. Flores, Gianluca Gilardoni, Omar Malagón

**Affiliations:** 1 Programa de Doctorado en Química, Universidad Técnica Particular de Loja (UTPL), Paris s/n y Praga, Loja 110107, Ecuador; drvinueza3@utpl.edu.ec; 2 Facultad de Ciencias, Escuela Superior Politécnica de Chimborazo (ESPOCH), Panamericana Sur Km 1½, Riobamba 060155, Ecuador; 3 Departamento de Química, Facultad de Ciencias, Campus Fuentenueva, Universidad de Granada, 18071 Granada, Spain; mariuxi.flores@unach.edu.ec; 4 Facultad de Ingeniería, Universidad Nacional de Chimborazo (UNACH), Av. Antonio José de Sucre Km 1½ vía a Guano, Riobamba 060155, Ecuador; 5 Departamento de Química y Producción, Universidad Técnica Particular de Loja (UTPL), Paris s/n y Praga, Loja 110107, Ecuador; ggilardoni@utpl.edu.ec

**Keywords:** *Aristolochia lingulata*, essential oil, enantioselective analysis, mass spectrometry, enzyme inhibition

## Abstract

Species of the genus *Aristolochia* are recognized as a rich source of structurally diverse specialized metabolites; however, the volatile chemical composition and biological properties of several Neotropical species remain poorly characterized. In particular, no information is currently available on the essential oil of *Aristolochia lingulata* Ule ex Pilg., which motivated this chemical, enantioselective, and preliminary biological investigation. The leaf essential oil was analyzed by GC-MS and GC-FID using nonpolar and polar stationary phases, while enantioselective GC-MS was employed to determine the distribution of selected chiral metabolites. Biological activity was evaluated through acetylcholinesterase, butyrylcholinesterase, pancreatic lipase, DPPH, and ABTS assays. A total of 57 constituents were identified and quantified, accounting for 93.5% and 91.3% of the oil on the two stationary phases. Sesquiterpene hydrocarbons and oxygenated sesquiterpenoids predominated, with dauca-5,8-diene, *trans*-(*E*)-nerolidol, β-chamigrene, β-cyclocitral, limonene, δ-amorphene, τ-muurolol, and manool among the main constituents. The oil showed moderate acetylcholinesterase inhibition (IC_50_ = 79.8 ± 0.3 µg/mL), whereas weaker inhibition was observed against butyrylcholinesterase (IC_50_ = 176.1 ± 3.3 µg/mL) and pancreatic lipase (IC_50_ = 190.8 ± 9.4 µg/mL). Radical scavenging activity was also weak in the DPPH and ABTS assays (SC_50_ = 849.7 ± 7.4 and 338.0 ± 3.9 µg/mL, respectively). Overall, this study provides the first characterization of the essential oil of *A. lingulata*, revealing a distinctive sesquiterpene-rich volatile profile and moderate enzyme-inhibitory activity. These findings expand the phytochemical knowledge of Neotropical *Aristolochia* species and provide a basis for further investigation of their bioactive constituents, mechanisms of action, and toxicological safety.

## 1. Introduction

The genus *Aristolochia* L. (Aristolochiaceae, Piperales) comprises several hundred species distributed predominantly in tropical and subtropical regions. Species of this genus have a long history of ethnobotanical use, particularly for gastrointestinal and inflammatory disorders and as traditional remedies for snake and other venomous animal bites [1,2,3,4,5]. In the Neotropics, *Aristolochia* is highly diverse, yet the phytochemical composition of many species remains insufficiently investigated. *Aristolochia lingulata* Ule ex Pilg. is a species native to Ecuador, Peru, and Bolivia, occurring mainly in tropical environments, for which information on volatile metabolites is currently very limited [2,6].

Phytochemical investigations of *Aristolochia* species have revealed a broad diversity of specialized metabolites, including alkaloids, lignans, flavonoids, terpenoids, and phenanthrene derivatives [4,7]. Among these metabolites, volatile constituents and essential oils have received comparatively less attention. Essential oils from different *Aristolochia* species have commonly been obtained by hydrodistillation and characterized by gas chromatography coupled with flame ionization or mass spectrometric detection. Previous investigations of species such as *A. elegans*, *A. mollissima*, *A. delavayi*, *A. trilobata*, and *A. fordiana* have revealed chemically diverse volatile profiles, frequently containing mono- and sesquiterpenoids, and have reported antimicrobial, cytotoxic, insecticidal, and other biological effects [8,9,10,11]. These findings suggest that the volatile fraction represents an additional and still incompletely explored dimension of the chemical diversity of the genus.

The investigation of *Aristolochia* species nevertheless requires particular toxicological caution. Several members of the genus contain aristolochic acids, especially aristolochic acids I and II, which are nitrophenanthrene carboxylic acids with well-established nephrotoxic, genotoxic, mutagenic, and carcinogenic effects [12,13]. Exposure to aristolochic acid-containing herbal products has been associated with aristolochic acid nephropathy, progressive renal failure, and an increased risk of upper urinary tract and other urothelial carcinomas. Consequently, regulatory authorities in several countries have restricted or prohibited medicinal products containing *Aristolochia* species or aristolochic acids [14,15]. Accordingly, the detection of biological activity in an *Aristolochia*-derived preparation should not be interpreted as evidence of therapeutic suitability or safety. Although essential oils primarily comprise volatile and semi-volatile compounds rather than the non-volatile aristolochic acids typically associated with these toxic effects, comprehensive safety assessment remains necessary before any potential application can be considered.

Essential oils are complex mixtures dominated by volatile constituents such as mono- and sesquiterpenes and their oxygenated derivatives, and their composition may vary according to species, plant organ, developmental stage, geographical origin, environmental conditions, and extraction procedure [16,17]. In *Aristolochia*, essential oil studies remain comparatively scarce, although reports on species such as *A. fordiana* have shown sesquiterpene- and monoterpene-rich profiles and preliminary biological activity, supporting the relevance of expanding volatile metabolite studies within the genus [18]. GC-FID and GC-MS are therefore widely employed for qualitative and quantitative characterization of these mixtures. In addition to their chemical diversity, essential oils have been investigated as sources of compounds capable of modulating biological targets, including enzymes related to oxidative stress, neurological processes, and lipid metabolism. Nevertheless, observed in vitro activities depend strongly on chemical composition, concentration, and experimental conditions and should be interpreted as preliminary evidence rather than direct indications of therapeutic efficacy.

Beyond conventional compositional analysis, enantioselective profiling can provide an additional level of chemical characterization because many terpenoid constituents of essential oils are chiral. Enantiomers may differ in sensory characteristics, biological interactions, and ecological functions, despite having identical molecular formulas and conventional mass spectra. Enantioselective gas chromatography can therefore complement conventional GC-MS analysis and provide information relevant to chemotaxonomy, authentication, and the characterization of natural enantiomeric patterns [19,20]. However, enantiomeric data remain scarce for essential oils from tropical *Aristolochia* species and, more broadly, from many poorly investigated Neotropical plants.

Despite previous studies on the volatile fractions of other *Aristolochia* species, no information is currently available on the essential oil composition, enantiomeric distribution, or associated biological properties of *A. lingulata*. This lack of information represents a relevant phytochemical gap, particularly considering the high botanical diversity of the Neotropics and the limited characterization of their aromatic plant resources. Therefore, the present study aimed to isolate the leaf essential oil of *A. lingulata*, characterize its chemical composition using GC-FID and GC-MS on stationary phases of different polarity, determine the enantiomeric distribution of selected chiral constituents by enantioselective GC-MS, and evaluate selected biological activities through preliminary in vitro assays. To the best of our knowledge, this is the first integrated chemical, enantioselective, and biological characterization of the essential oil of *A. lingulata*, providing new information on the volatile chemodiversity of Neotropical *Aristolochia* while establishing a basis for subsequent investigations of individual bioactive constituents, mechanisms of action, and toxicological safety.

## 2. Results

### 2.1. Chemical Composition of Essential Oil

The aerial parts of *A. lingulata* Ule ex Pilg. were subjected to steam distillation, yielding an essential oil (EO) with a yield of 0.03% (*w*/*w*) based on dry plant material. A total of fifty-seven compounds were identified and quantified by GC-MS and GC-FID analyses using two capillary columns of different polarity. These compounds accounted for 93.5% and 91.3% of the total EO composition on the nonpolar and polar columns, respectively. The main compounds (≥3% on both columns) identified in the volatile fraction were limonene (4.4–4.1%, **6**), sorbic acid (4.6–3.9%, **10**), β-cyclocitral (4.8–4.5%, **12**), cadina-1(6),4-diene (3.8–3.8%, **25**), dauca-5,8-diene (11.1–10.9%, **26**), β-chamigrene (5.0–5.0%, **28**), epizonarene (3.5–3.6%, **31**), *trans*-(*E*)-nerolidol (11.3–11.8%, **37**), τ-muurolol (3.7–3.4%, **49**) and manool (4.4–4.6%, **55**). The results are summarized in Table 1.

**Figure 1:**
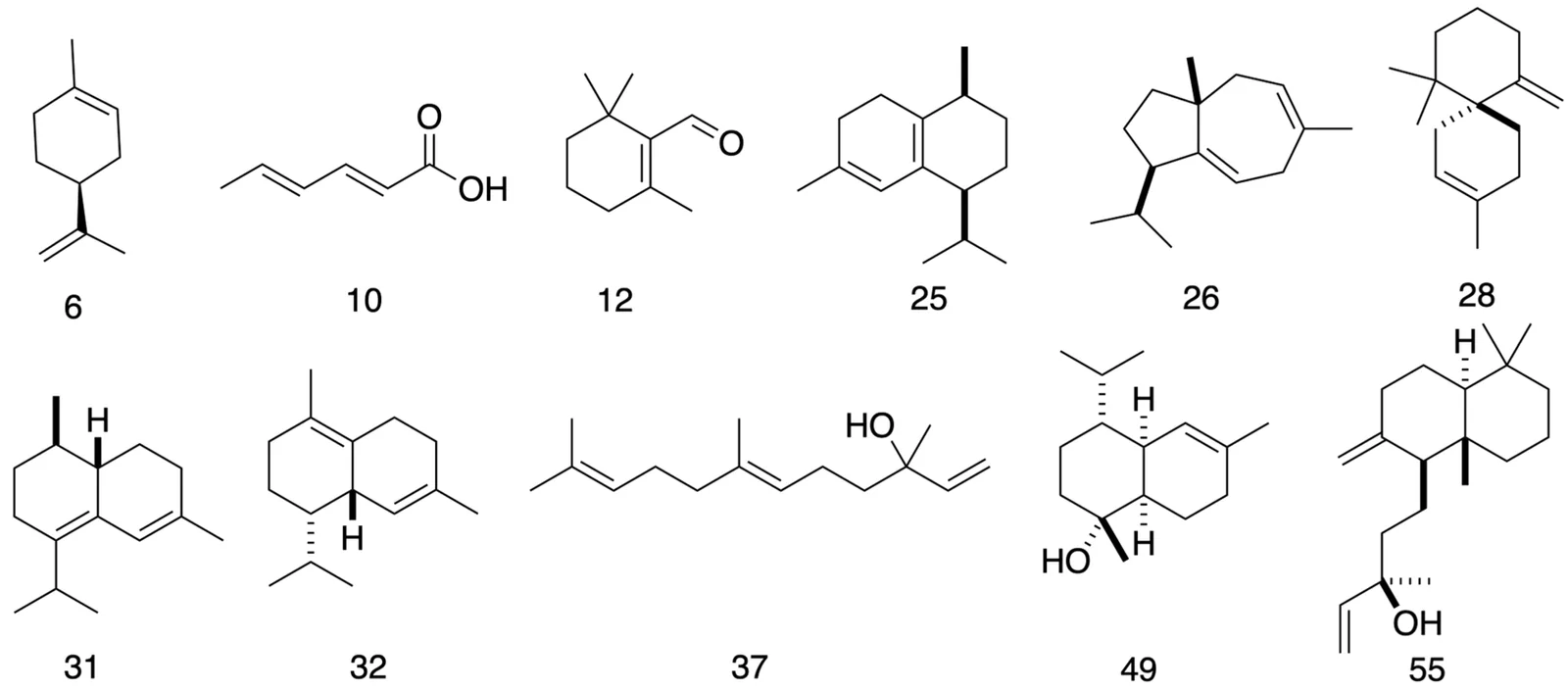
The major components (≥3.0% on at least one column) of *A. lingulata* Ule ex Pilg. essential oil. The numbers refer to Table 1: (*S*)-(−)-limonene (**6**), sorbic acid (**10**), β-cyclocitral (**12**), cadina-1(6),4-diene (**25**), dauca-5,8-diene (**26**), β-chamigrene (**28**), epizonarene (**31**), δ-amorphene (**32**), *trans*-(*E*)-nerolidol (**37**), τ-muurolol (**49**), and manool (**55**).

**Figure 2:**
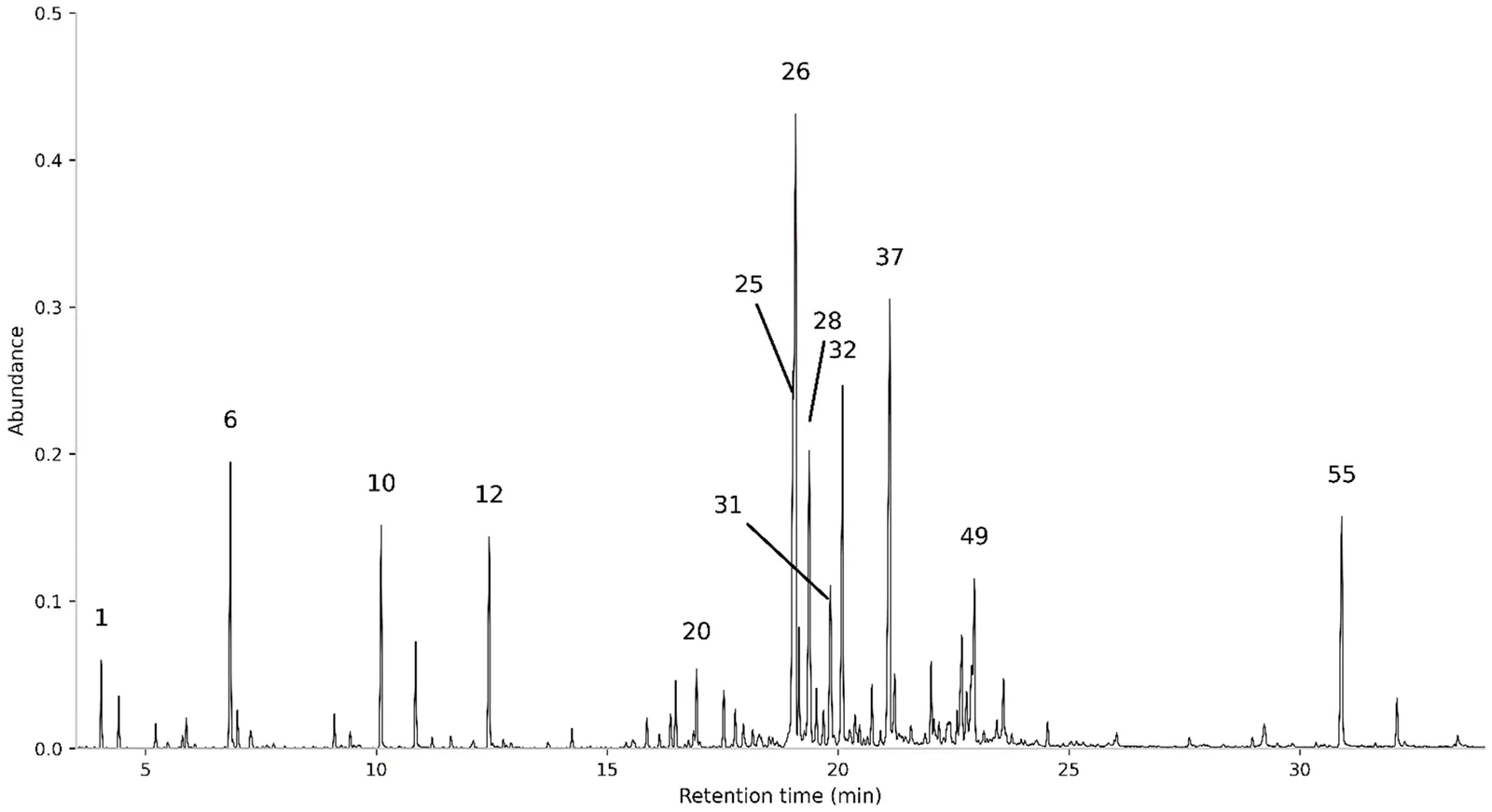
GC-MS profile of *A. lingulata* Ule ex Pilg. essential oil on 5%-phenyl-methyl-polysiloxane stationary phase. Peak numbers correspond to the sequential numbering used in Table 1.

**Figure 3:**
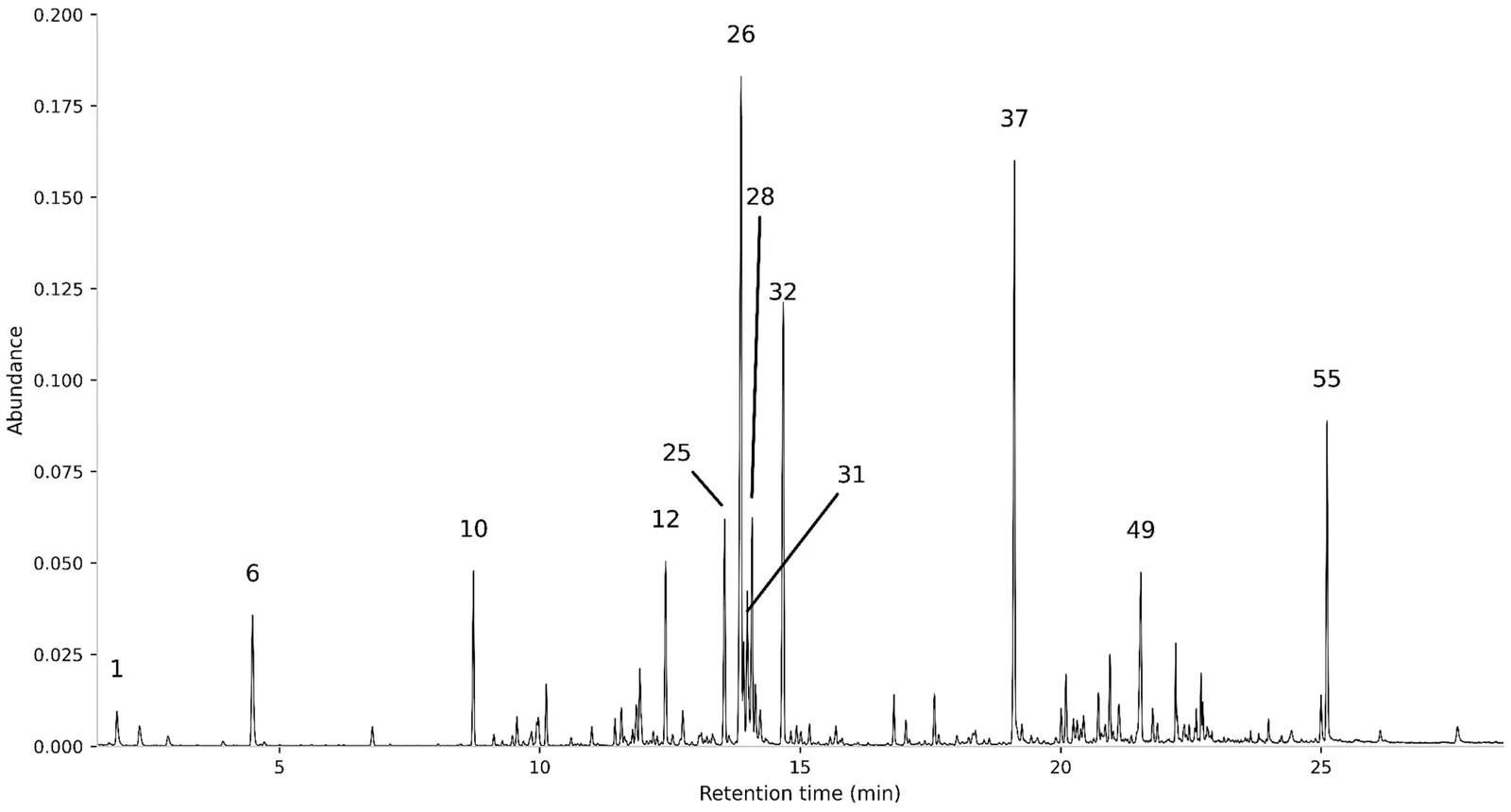
GC-MS profile of *A. lingulata* Ule ex Pilg. essential oil on polyethylene glycol stationary phase. Peak numbers correspond to the sequential numbering used in Table 1.

The chemical profile of the essential oil of *A. lingulata* Ule ex Pilg. was dominated by sesquiterpene hydrocarbons, which accounted for 36.2–36.8% of the total composition, followed by oxygenated sesquiterpenes with 25.3–23.7%. Minor but notable contributions were observed for oxygenated monoterpenes (7.7–7.0%) and monoterpene hydrocarbons (6.8–6.5%), whereas oxygenated diterpenes were present in comparatively lower proportions (4.4–4.6%). The remaining identified constituents, grouped as “others”, represented 13.1–12.7%. The chemical structures of the major constituents (≥3.0% on at least one column) are shown in Figure 1, whereas the chromatograms obtained on the nonpolar and polar stationary phases are shown in Figure 2 and Figure 3, respectively.

### 2.2. Enantioselective Analysis

An enantioselective GC-MS analysis was carried out on the essential oil of *A. lingulata* Ule ex Pilg. (Table 2). Three compounds, (1*S*,5*S*)-(–)-α-pinene, (1*S*,5*S*)-(–)-β-pinene and (*S*)-(–)-limonene, were enantiomerically pure, whereas camphene occurred as both enantiomers in a scalemic mixture. The enantiomeric purity of α-pinene was determined on a 2,3-diacetyl-6-*tert*-butyldimethylsilyl-β-cyclodextrin-based column, because its enantiomers are inseparable on the other chiral selector. For all other chiral compounds, a 2,3-diethyl-6-*tert*-butyldimethylsilyl-β-cyclodextrin-based column was used because it provided better separation. The complete results are detailed in Table 2.

### 2.3. Cholinesterase Inhibition

**Figure 4:**
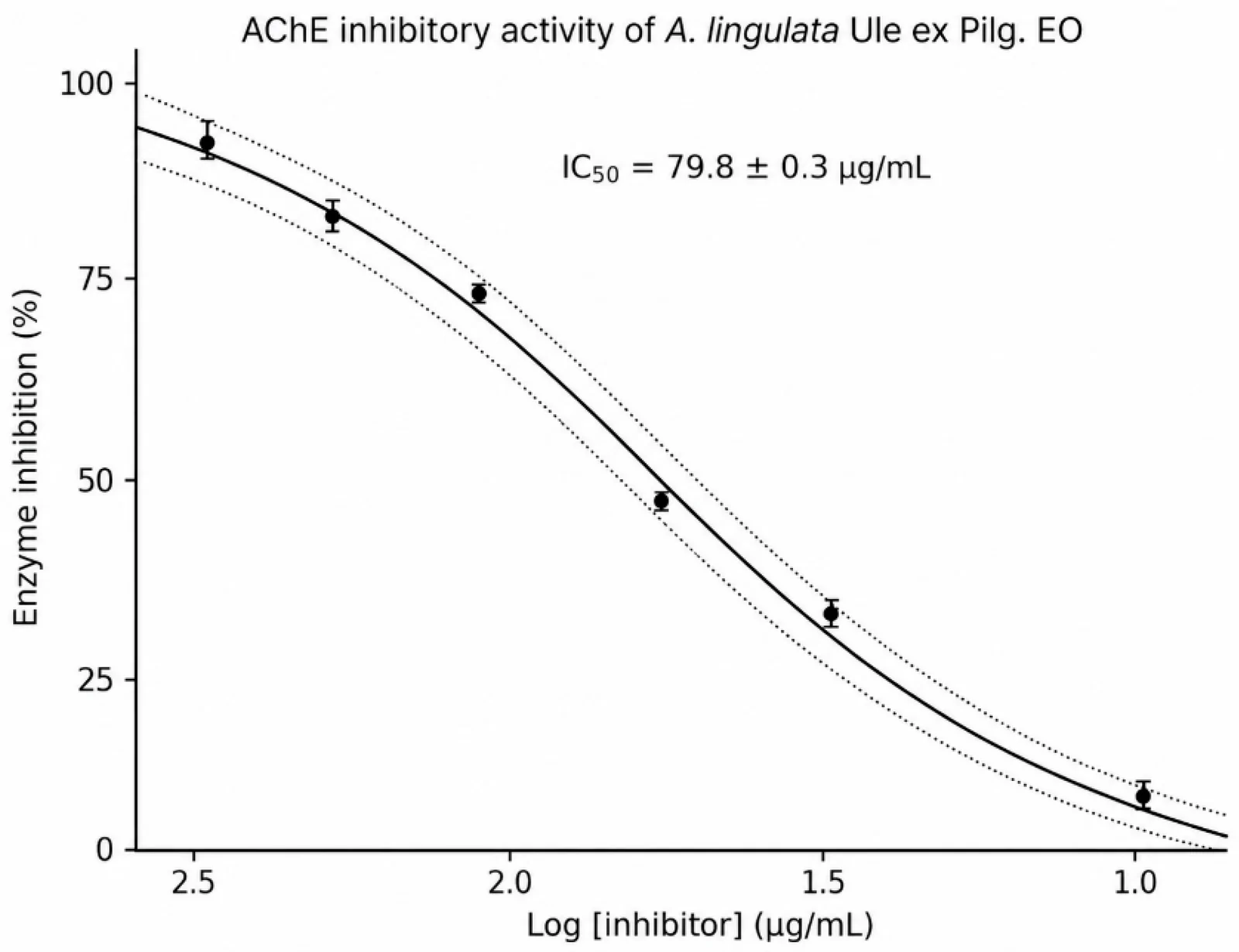

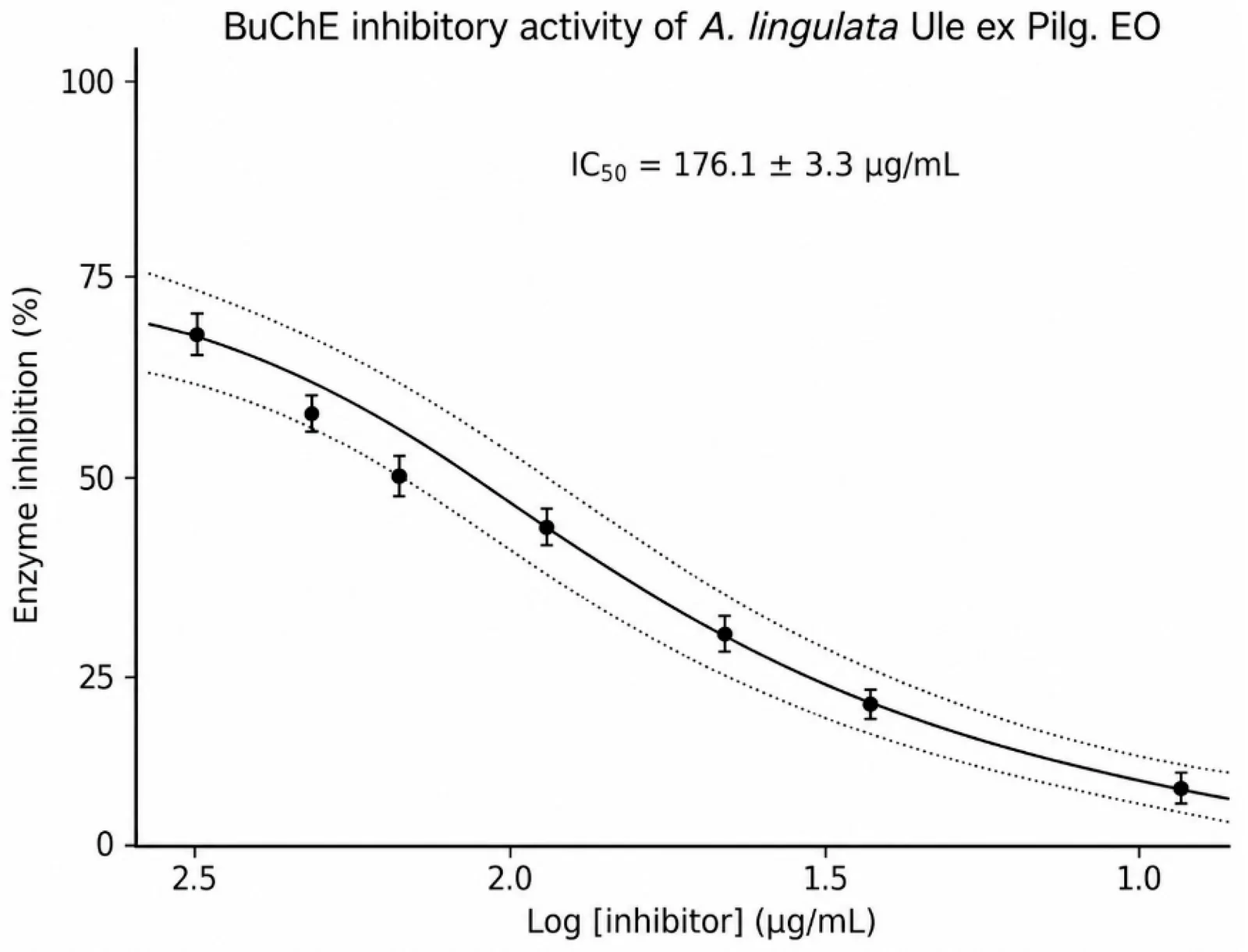
Inhibition of cholinesterases (acetylcholinesterase and butyrylcholinesterase) from *A. lingulata* Ule ex Pilg. essential oil expressed as IC_50_ ± SD, *n* = 3. The solid line represents the fitted dose–response curve, while the dashed lines indicate the 95% confidence interval of the fitted curve.

The AChE inhibitory effect is relevant due to the role of this enzyme in acetylcholine hydrolysis and its pharmacological importance in neurodegenerative disorders such as Alzheimer’s disease. AChE is also a key neurophysiological target in insects, where its inhibition can disrupt cholinergic neurotransmission and contribute to paralysis and mortality. Accordingly, several essential oils and terpenoid constituents have been investigated as natural insecticidal agents, with AChE inhibition proposed as one of their possible mechanisms of action. However, these effects are generally multifactorial and may also involve octopaminergic signaling, GABA-gated ion channels, and other neurophysiological targets [48,49,50]. The anticholinesterase activity of *A. lingulata* Ule ex Pilg. essential oil was determined spectrophotometrically using the modified Ellman method. The essential oil showed moderate inhibitory activity against acetylcholinesterase (AChE), with an IC_50_ value of 79.8 ± 0.3 µg/mL (Figure 4). In comparison, the positive control galantamine showed stronger inhibitory activity, with an IC_50_ value of 1.2 ± 0.3 µg/mL. Although the essential oil was less potent than the reference drug, it showed measurable AChE inhibition, indicating a preliminary interaction with this enzyme. This effect may be associated with its terpene-rich composition, particularly the presence of sesquiterpene hydrocarbons, oxygenated sesquiterpenes, and minor monoterpenes, although the contribution of individual constituents remains to be established.

In the BuChE inhibition assay, the essential oil of *A. lingulata* Ule ex Pilg. showed a concentration-dependent response, with an IC_50_ value of 176.1 ± 3.3 µg/mL (Figure 4). This activity was considerably weaker than that of galantamine (58.9 ± 1.5 µg/mL), used as the positive control. Accordingly, the BuChE inhibition should be regarded as a preliminary and relatively weak biological effect. Together with the AChE results, these data indicate differential inhibitory activity toward the two cholinesterases, although the contribution of individual volatile constituents remains to be determined.

### 2.4. Lipase Inhibition

**Figure 5:**
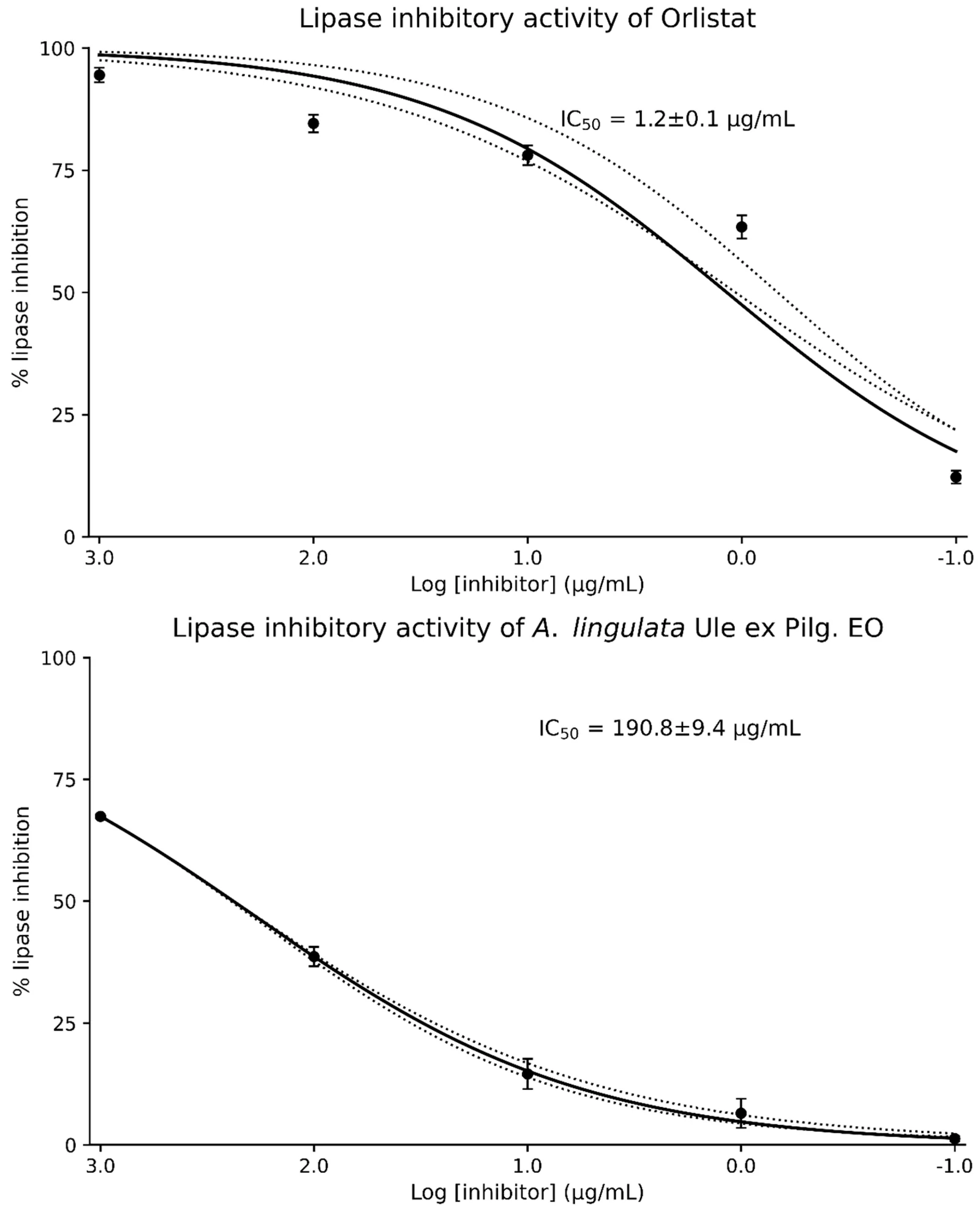
Inhibition of lipase from orlistat and *A. lingulata* Ule ex Pilg. essential oil expressed as IC_50_ ± SD, *n* = 3. The solid line represents the fitted dose–response curve, while the dashed lines indicate the 95% confidence interval of the fitted curve.

The pancreatic lipase inhibitory activity of *A. lingulata* Ule ex Pilg. essential oil (EO) was evaluated using orlistat as the positive control (Figure 5). Both samples showed a concentration-dependent inhibitory profile within the tested range of 0.1–1000 µg/mL. Orlistat exhibited strong inhibition, reaching 94.5 ± 1.6% at 1000 µg/mL and maintaining high activity at 100 and 10 µg/mL, with inhibition values of 84.6 ± 2.8% and 78.1 ± 2.5%, respectively. At the lowest concentration tested, 0.1 µg/mL, the inhibition decreased to 12.2 ± 1.9%. The calculated IC_50_ value for orlistat was 1.2 ± 0.1 µg/mL, confirming the suitability of the assay and the expected potency of this reference inhibitor.

The EO of *A. lingulata* Ule ex Pilg. also inhibited pancreatic lipase in a dose-dependent manner, although with lower potency than orlistat. At 1000 µg/mL, the EO produced 67.4 ± 0.4% inhibition, while at 100 µg/mL the inhibition decreased to 38.7 ± 2.0%. Lower concentrations showed weaker activity, with inhibition values of 14.6 ± 3.1%, 6.5 ± 3.0%, and 1.3 ± 0.5% at 10, 1, and 0.1 µg/mL, respectively. The calculated IC_50_ value was 190.8 ± 9.4 µg/mL, indicating a moderate pancreatic lipase inhibitory effect. This activity is considerably weaker than that of orlistat, but it is relevant for a chemically complex essential oil dominated by volatile terpenoids rather than highly polar phenolic or saponin-like constituents.

### 2.5. Total Antioxidant Capacity

The total antioxidant scavenging capacity of the EO was assessed using the DPPH and ABTS models. Table 3 shows the scavenging capacity (SC_50_) of the EO and the positive control. The concentration–response curve was constructed using a range up to 1000 µg/mL, which was sufficient to determine the SC_50_ value.

The antioxidant activity of *Aristolochia lingulata* Ule ex Pilg. essential oil was evaluated using two radical scavenging models, DPPH and ABTS. The essential oil showed SC_50_ values of 849.7 ± 7.4 µg/mL and 338.0 ± 3.9 µg/mL for DPPH and ABTS, respectively, whereas Trolox, used as the positive control, showed markedly stronger activity, with SC_50_ values of 13.5 ± 1.2 µg/mL and 11.8 ± 1.2 µg/mL, respectively. These results indicate that the essential oil has a low radical scavenging capacity, particularly in the DPPH assay. Since lower SC_50_ or IC_50_ values indicate stronger antioxidant activity, the high SC_50_ values obtained for the essential oil suggest limited direct free radical scavenging ability compared with Trolox [51].

## 3. Discussion

### 3.1. Chemical and Enantiomeric Composition and Chemotaxonomic Significance

This chemical profile is consistent with the trend observed in numerous species of the genus Aristolochia, whose essential oils are typically dominated by sesquiterpenes and exhibit high interspecific variability. A comprehensive review of the genus notes that Aristolochia EOs are generally characterized by high proportions of sesquiterpenes and the absence of a universal chemical pattern, reflecting marked chemotaxonomic diversity among species [4]. Furthermore, a comparative study of five species endemic to Turkey showed that sesquiterpenes constitute the predominant group in most essential oils of the genus, although the specific compositions vary by taxon [52].

A comparison with previously reported Aristolochia species revealed marked differences in essential oil composition. In A. triangularis, the predominant constituents included germacrene D, (*E*)-nerolidol, bicyclogermacrene, β-elemene, (*E*)-β-caryophyllene, and germacrene A, depending on the plant organ analyzed. (*E*)-nerolidol reached 17.89% in stem essential oils, a concentration comparable to that detected in *A. lingulata* Ule ex Pilg. However, dauca-5,8-diene and β-chamigrene, identified as major constituents of *A. lingulata* Ule ex Pilg., were not reported among the dominant compounds of *A. triangularis* [53]. Such differences emphasize the remarkable chemical variability of essential oils across species of the genus *Aristolochia*.

Similarly, the composition observed in *A. lingulata* Ule ex Pilg. differs considerably from that described for *A. argentina*, whose essential oils are characterized by exceptionally high concentrations of argentilactone (57–89%) and a relatively lower proportion of terpenes, with bicyclogermacrene predominating in the aerial parts in the underground organs [54]. These differences highlight the remarkable chemical diversity within the genus *Aristolochia* and suggest that South American species have developed distinct metabolic profiles possibly associated with ecological, evolutionary, or physiological factors.

The significant presence of β-chamigrene in *A. lingulata* Ule ex Pilg. is particularly interesting because this compound has also been reported as the main constituent of the essential oil of *A. fordiana*, where it accounted for approximately 17% of the total composition. In that study, the essential oil exhibited antibacterial activity against *Staphylococcus aureus* and *Bacillus subtilis*, as well as moderate cytotoxic activity against human tumor cell lines [18]. Although the observed bioactivity cannot be directly attributed to a single metabolite, the presence of *β*-chamigrene in both species suggests that this sesquiterpene may contribute to the biological potential of the essential oils of this genus. Furthermore, compounds such as limonene, *β*-cyclocitral, *trans*-(*E*)-nerolidol, and *τ*-muurolol have been widely recognized for their biological activities. Limonene is one of the most studied monoterpenes and has been associated with insecticidal and repellent activities. In *A. trilobata*, where this compound is one of the main constituents of the essential oil, toxic and repellent effects were observed against termites and leaf-cutting ants. Similarly, *trans*-(*E*)-nerolidol has been reported in numerous bioactive essential oils due to its antimicrobial, antioxidant, and insecticidal properties; therefore, its high abundance in *A. lingulata* Ule ex Pilg. could contribute significantly to the biological activity of this essential oil [55].

The enantioselective analysis revealed a highly selective stereochemical profile, with α-pinene, β-pinene, and limonene detected as enantiomerically pure, whereas camphene occurred as a scalemic mixture. This pattern suggests compound-specific enzymatic control during monoterpene biosynthesis and may provide useful chemotaxonomic and authenticity markers for *A. lingulata* essential oil. However, because the oil was obtained from a single collection and distillation, additional samples from different populations and seasons are required to determine whether this enantiomeric profile is stable within the species.

### 3.2. Enzyme-Inhibitory and Radical Scavenging Activities

#### 3.2.1. Cholinesterase Inhibition

The essential oil of *A. lingulata* Ule ex Pilg. showed measurable cholinesterase inhibition, with greater activity against AChE (IC_50_ = 79.8 ± 0.3 µg/mL) than against BuChE (IC_50_ = 176.1 ± 3.3 µg/mL). However, the oil was substantially less potent than galantamine, particularly against AChE, and these results should therefore be interpreted as preliminary screening data rather than evidence of therapeutic potential. AChE inhibition remains pharmacologically relevant in the symptomatic management of Alzheimer’s disease, whereas BuChE may also contribute to cholinergic regulation during disease progression [56,57]. Nevertheless, the present results indicate only a measurable interaction of the essential oil with these enzymes under the experimental conditions employed.

The approximately 2.2-fold difference between the AChE and BuChE IC_50_ values suggests differential inhibition of the two cholinesterases. Similar variations in cholinesterase selectivity have been described for other essential oils, reflecting differences in their chemical composition [58,59,60]. However, direct comparison among studies should be made cautiously because enzyme source, substrate, incubation conditions, and essential oil composition can substantially influence the measured inhibitory response.

The terpene-rich composition of the oil may contribute to the observed cholinesterase inhibition, since mono- and sesquiterpenoids have previously been reported to interact with cholinesterases [56]. Nevertheless, any relationship between individual constituents and the activity observed here remains hypothetical. Compounds such as *trans*-(*E*)-nerolidol, β-chamigrene, τ-muurolol, spathulenol, and other terpenoids were not tested individually, and no fractionation or mechanistic studies were performed. Therefore, neither individual activity nor synergistic or additive effects can be established from the present data.

Overall, the cholinesterase results should be regarded as exploratory evidence of enzyme inhibition by the volatile mixture. Further studies using isolated constituents, fractionated samples, kinetic approaches, and independent biological replicates would be required to determine the compounds responsible for the observed effects and their mechanisms of interaction.

#### 3.2.2. Lipase Inhibition

The essential oil of *A. lingulata* Ule ex Pilg. inhibited pancreatic lipase with an IC_50_ of 190.8 ± 9.4 µg/mL, whereas orlistat showed substantially greater activity (IC_50_ = 1.2 ± 0.1 µg/mL). Thus, under the present experimental conditions, the essential oil displayed only moderate inhibitory activity and was approximately 160-fold less potent than the reference compound. This result should therefore be considered part of a preliminary biological screening rather than evidence of pharmacological applicability.

Pancreatic lipase inhibition has also been reported for other essential oils, although considerable variation exists among species and experimental systems [61,62,63,64]. The activity observed for *A. lingulata* falls within the broad range described for relatively weak or moderate natural lipase inhibitors. However, comparisons among studies should be interpreted cautiously because differences in enzyme source, substrate, solvent system, sample solubility, and assay conditions may markedly affect IC_50_ values.

The predominance of sesquiterpene hydrocarbons and oxygenated sesquiterpenes in the oil provides a possible chemical basis for the observed inhibition, but this association remains speculative. Previous studies have suggested that sesquiterpenes may contribute to pancreatic lipase inhibition [61]; however, the individual constituents of *A. lingulata* EO were not evaluated separately. Consequently, the possible contribution of compounds such as *trans*-(*E*)-nerolidol, spathulenol, τ-muurolol, eremoligenol, hinesol, or manool should be regarded only as a working hypothesis requiring experimental confirmation.

Taken together, the lipase assay indicates measurable but modest inhibition by the essential oil. Additional studies involving isolated constituents, fractionation, enzyme kinetics, appropriate controls for sample solubility and interference, and independent plant material would be required before mechanistic or practical conclusions can be drawn.

#### 3.2.3. Total Antioxidant Capacity

The essential oil showed weak radical scavenging activity in both assays, with SC_50_ values of 849.7 ± 7.4 µg/mL for DPPH and 338.0 ± 3.9 µg/mL for ABTS. The lower SC_50_ obtained with ABTS indicates a comparatively better response in this assay, although the activity remained substantially weaker than that of Trolox. This difference may reflect the greater compatibility of ABTS with lipophilic constituents, whereas DPPH responses can be more affected by steric accessibility, reaction kinetics, and solubility limitations [65,66].

The weak activity is consistent with the terpene-rich and largely nonphenolic composition of the essential oil. Essential oils dominated by hydrocarbon terpenes and nonphenolic oxygenated terpenoids often display lower direct radical scavenging activity than oils rich in phenolic constituents such as thymol, carvacrol, or eugenol [67,68,69]. Accordingly, the present results indicate that the volatile fraction of *A. lingulata* is not an efficient direct radical scavenger under the conditions tested.

Although essential oils may influence oxidative processes through mechanisms not captured by DPPH or ABTS assays [67,68,69,70,71,72], such effects were not evaluated in the present study. Therefore, the current findings should be restricted to direct radical scavenging capacity and should not be extrapolated to antioxidant effects in biological systems.

Overall, the DPPH and ABTS results indicate limited radical scavenging capacity and should be regarded as preliminary screening data complementing the chemical characterization of the essential oil.

#### 3.2.4. Study Limitations and Toxicological Considerations

An important limitation of the present study is that the essential oil was obtained from a single plant collection and a single distillation. Essential oil composition can vary according to geographical origin, environmental conditions, season, phenological stage, plant age, and other biological and ecological factors. Consequently, the chemical profile reported here should not be considered representative of *A. lingulata* as a species or interpreted as evidence of a stable chemotype without analysis of additional populations, collection periods, and independent distillations. The same limitation applies to the biological assays, since the measured cholinesterase, lipase, and radical scavenging activities reflect only the composition of the particular essential oil sample investigated.

Toxicological considerations are especially important for species of *Aristolochia*. Aristolochic acids are well-established nephrotoxic, genotoxic, and carcinogenic compounds associated with severe renal injury and urothelial malignancies, and their occurrence has resulted in substantial restrictions on the medicinal use of *Aristolochia*-derived products [73]. Although aristolochic acids are non-volatile compounds and would not normally be expected to constitute typical components of a steam-distilled essential oil, their absence was not specifically demonstrated in the present investigation. Therefore, the biological activities reported here should not be interpreted as evidence of safety or support for medicinal use.

Future investigations should include targeted analytical methods capable of confirming the absence of aristolochic acids and other potentially hazardous constituents, together with cytotoxicity and broader toxicological evaluation. Such studies are essential before any possible practical application of the essential oil can be considered.

Taken together, the present biological results should be interpreted as exploratory observations that complement the first chemical and enantioselective characterization of *A. lingulata* essential oil. They provide a basis for generating hypotheses for future chemical and biological studies but do not establish pharmacological efficacy, safety, or immediate translational potential.

## 4. Materials and Methods

### 4.1. Plant Collection and Extraction

The leaves of *A. lingulata* Ule ex Pilg. were collected on 10 October 2025 in Santa Rosa (Tena Canton, Napo Province, Ecuador) at 496 m above sea level. Plant material was obtained from shrubs scattered around the coordinates 1°04′57″ S and 77°53′27″ W, within an approximated 200 m radius; a composite average sample was obtained and mixed for analysis. Samples were immediately transported in sterile containers to Bioproducts Plant at the Universidad Técnica Particular de Loja (UTPL) for processing. Botanical identification was carried out by Professor Nixon Cumbicus, finding whether they matched in morphological aspects with reference specimens. The leaves were dried in an oven at 35 °C for 48 h to minimize evaporation, reduce moisture, and prevent thermal degradation of the volatile fraction prior distillation. A voucher specimen was assigned under the code HUTPL 16372 and deposited at UTPL Herbarium. This study was conducted under research permit No MAATE-ARSFC-2025-0167 granted by the Ministerio de Ambiente, Agua y Transición Ecológica del Ecuador (MAATE). The botanical sample was transported to the laboratories of UTPL under the mobilization guide MAATE-CMARG-2025-0064 issued by the MAATE.

### 4.2. Essential Oil Distillation and Sample Preparation

Steam distillation was performed using modified Dean–Stark apparatus [74]. Briefly, the entire dried leaves of *A. lingulata* Ule ex Pilg. (400 g) were distilled for 7 h according to the literature [75,76]. This process was carried out only once using all the collected plant material, due difficult access to the collection site. To remove excess water, the essential oil was subjected to treatment with anhydrous sodium sulfate (Sigma-Aldrich, St. Louis, MO, USA) and permanently stored at −15 °C until use. For chromatographic analysis (GC), four samples were prepared by weighing 10 µL aliquots and diluting them with 1 mL of cyclohexane, which contained *n*-nonane as an internal standard at a concentration of 0.70 mg/mL.

### 4.3. Qualitative Chemical Analysis

The chemical profile of the volatile components of *A. lingulata* Ule ex Pilg. EO was qualitatively characterized by gas chromatography–mass spectrometry (GC-MS) using a Trace 1310 gas chromatograph coupled to an ISQ 7000 single-quadrupole mass spectrometer (Thermo Fisher Scientific, Waltham, MA, USA). A volume of 1 μL of the essential oil solution in cyclohexane was injected in split mode (40:1), using helium (Indura S.A., Guayaquil, Ecuador) as the carrier gas at a constant flow rate of 1 mL/min. The injector temperature was maintained at 250 °C. Electron-impact ionization was performed at 70 eV, and mass spectra were acquired in SCAN mode over an *m*/*z* range of 40–400.

Separations were performed on two stationary phases of markedly different polarity: a non-polar DB-5ms column (5% phenylmethylpolysiloxane) and a polar TR-WAX column (polyethylene glycol), both measuring 30 m × 0.25 mm i.d., with a film thickness of 0.25 μm (Thermo Fisher Scientific, Waltham, MA, USA). The use of two stationary phases provided complementary chromatographic selectivity, which is particularly relevant for essential oils because structurally related terpenes may exhibit similar electron-impact mass spectra and closely related retention behavior, potentially leading to partial coelution or ambiguous identification on a single column. The DB-5ms phase was particularly suitable for resolving hydrocarbon mono- and sesquiterpenes and for comparison with widely available retention index data obtained on 5%-phenyl-polysiloxane phases. In contrast, the polar PEG phase modified the retention behavior of oxygenated and more polar volatile constituents and provided additional selectivity for compounds that could not be unequivocally differentiated on the non-polar phase. Therefore, chromatographic analysis on both phases, together with comparison of mass spectra and linear retention indices, increased the reliability of compound identification.

The oven temperature program for the DB-5ms column was as follows: 60 °C (5 min), ramped to 100 °C, then increased at 3 °C/min to 150 °C, followed by 5 °C/min to 200 °C and finally 15 °C/min to 250 °C, with a 15 min hold at the final temperature. The same temperature program was applied to the TR-WAX column, except that the final temperature was limited to 230 °C and maintained for 15 min.

The identification of compounds in the essential oil of *A. lingulata* Ule ex Pilg. was based on comparison of their mass spectra and linear retention indices (LRIs) with reference data. LRIs were calculated using a homologous series of *n*-alkanes (C9–C25) according to the method of Van Den Dool and Kratz [77]. The *n*-alkanes were purchased from Sigma-Aldrich (St. Louis, MO, USA). For each identified compound, the mean retention time was also calculated.

### 4.4. GC-FID Quantitative Analysis

Quantification of the constituents of the essential oil of *A. lingulata* Ule ex Pilg. was performed by gas chromatography with flame ionization detection (GC-FID). GC-FID and GC-MS measurements were conducted as separate chromatographic analyses; therefore, no post-column splitting or simultaneous acquisition by FID and MS was employed. For GC-FID quantification, chromatographic conditions equivalent to those used for the qualitative GC-MS analysis were applied, including the same stationary phases of different polarity (DB-5ms and TR-WAX), oven temperature programs, helium carrier gas flow rate (1 mL/min), and injector temperature (250 °C). The injection split ratio was adjusted to 10:1, compared with 40:1 for GC-MS analysis, to improve the detection and quantification of minor constituents.

The relative response factor (RRF) of each compound was calculated according to its enthalpy of combustion [78], and the integrated peak areas were corrected using the corresponding RRFs. This approach is based on a previously established and externally validated relationship between FID response and combustion enthalpy for volatile compounds and has been proposed as a practical alternative when authentic standards are not available for all analytes. Previous validation studies involving large datasets of volatile compounds have demonstrated good agreement between predicted and experimentally determined RRF values, and the approach has been incorporated into recommended practices for GC-FID quantification of volatile substances [79,80,81]. Accordingly, the present study applied an established predicted-RRF methodology rather than performing compound-specific calibration for each individual constituent. The resulting concentrations should therefore be interpreted within the expected uncertainty associated with predicted RRF-based quantification.

Two calibration curves, one for each stationary phase, were constructed using isopropyl caproate as the calibration standard and n-nonane as the internal standard (Sigma-Aldrich, St. Louis, MO, USA). Isopropyl caproate was synthesized and purified in this work, and its purity (98.8%) was confirmed by gas chromatographic analysis. Standard solutions were prepared according to the procedure described in the literature [82], and correlation coefficients greater than 0.999 were obtained for both calibration curves.

### 4.5. Enantioselective Analysis

An enantioselective analysis of the four pairs of enantiomers presented by the essential oil of *A. lingulata* Ule ex Pilg. was carried out using gas chromatography coupled to mass spectrometry (GC-MS). The analysis was performed using enantioselective capillary columns of 25 m length, 0.25 mm internal diameter and 0.25 μm phase thickness, with stationary phases based on β-cyclodextrin derivatives: 2,3-diacetyl-6-*tert*-butyldimethylsilyl-β-cyclodextrin (DAC) and 2,3-diethyl-6-*tert*-butyldimethylsilyl-β-cyclodextrin (DET) (Mega, Milan, Italy).

The instrumental conditions were as follows: a split ratio of 50:1, a constant helium carrier gas pressure of 70 kPa, and a temperature program commencing at 60 °C for 2 min, increasing at 2 °C/min until reaching 220 °C, and maintaining this final temperature for 2 min. Mass spectra and linear retention indices (LRIs), calculated using a series of C9–C25 *n*-alkanes (Sigma-Aldrich, St. Louis, MO, USA), were used to compare the detected enantiomers with data from the injection of enantiomerically pure standards. This was performed according to the method of Van den Dool and Kratz. The selection of the DAC and DET columns was based on their observed efficiency in resolving the specific enantiomeric pairs present in the sample.

### 4.6. AChE and BuChE Inhibitory Activity

Cholinesterase-inhibitory activities were determined according to Ellman et al. [83] with some modifications by López and co-workers [84]. Stock solutions with 600 U of AChE from *Electrophorus electricus* (Merck, Darmstadt, Germany) and BuChE from equine serum (Merck, Darmstadt, Germany) were prepared and kept at −20 °C. Acetylthiocholine iodide (ATCI), S-butyrylthiocholine iodide (BTCI), and 5,5′-dithiobis (2-nitrobenzoic) acid (DTNB) were obtained from Merck (Darmstadt, Germany). A volume of 50 µL of AChE or BuChE (both enzymes used at 6.0 U) in phosphate buffer (8 mM K_2_HPO_4_, 2.3 mM NaH_2_PO_4_, 0.15 NaCl, pH 7.5) and 50 µL of the sample dissolved in the same buffer and DMSO (final concentration 0.5%) were added to the wells. The plates were incubated for 30 min at room temperature. Then, 100 µL of the substrate solution (0.1 M Na_2_HPO_4_, 0.5 M DTNB, and 0.6 mM ATCI or 0.24 mM BTCI in Millipore water, pH 7.5) was added. These reagents were obtained from Merck (Darmstadt, Germany). After 10 min, the absorbance was read at 405 nm in a Multiskan^TM^ GO microplate spectrophotometer controlled with SkanIt^TM^ Software version 3.2 (Thermo Fisher Scientific, Vantaa, Finland). Enzymes activities were calculated as percent compared to a control using a buffer without any inhibitor. Galantamine was used as a positive control [85]. Initially, the inhibitory activity of the essential oil was assessed at 10, 100, and 200 µg/mL against both cholinesterases. Subsequently, concentration–response curves were constructed using essential oil concentrations of 10, 30, 50, 100, 150, 200, and 300 µg/mL to determine IC_50_ values against AChE and BuChE.

### 4.7. Lipase Inhibition

The activity was measured in 96-well microplates using previous protocols [86]. Each well contained 20 µL essential oil dilution or reference inhibitor (orlistat) at different concentrations and 160 µL of 2.5 mg/mL lipase, prepared in 100 mM Tris and 5 mM CaCl_2_ buffer, pH 7.0. After 15 min of preincubation, 20 µL of 10 mM pNPB solution was added to each well for another 15 min of incubation at 37 °C. Controls were performed to obtain 100% activity, containing buffer instead of samples or inhibitors. Blanks were also performed to avoid background interference, containing buffer instead of lipase. Absorbance was read at 405 nm, and orlistat was used as a reference inhibitor [87].

### 4.8. Total Antioxidant Capacity

The antioxidant capacity of the essential oil was evaluated using the DPPH and ABTS radical scavenging assays, following the methods described by Brand-Williams et al. [88]. and Re et al. [89], respectively, with slight modifications [90]. The radical scavenging activity was calculated from the absorbance values and expressed as percentage inhibition ± standard deviation. The essential oil was diluted in methanol to obtain concentrations ranging from 8 to 1000 μg/mL [91].

For the DPPH assay, a 0.1 mM solution of 2,2-diphenyl-1-picrylhydrazyl (DPPH) in methanol was prepared and adjusted to an absorbance of 1.0 ± 0.1 at 517 nm. Briefly, 20 µL of each essential oil dilution was mixed with 130 µL of the DPPH solution in a 96-well microplate. After incubation for 30 min at room temperature in the dark, the absorbance was measured at 517 nm.

For the ABTS assay, a 7.5 mM aqueous solution of 2,2′-azino-bis(3-ethylbenzothiazoline-6-sulfonic acid) was prepared. The ABTS radical cation (ABTS^•+^) was generated by reaction of the ABTS solution with 2.5 mM potassium persulfate, and the mixture was allowed to stand in the dark at room temperature for 16 h before use. Prior to the assay, the ABTS^•+^ stock solution was diluted with methanol to obtain an absorbance of 0.90 ± 0.05 at 734 nm. Then, 130 µL of the diluted ABTS^•+^ solution was mixed with 20 µL of each essential oil dilution in a 96-well microplate, and the absorbance was measured at 734 nm at 4 min after sample addition.

All biological assays were performed in triplicate (*n* = 3), and IC_50_ or SC_50_ values were calculated from the corresponding concentration–response curves and expressed as the mean ± standard deviation.

## 5. Conclusions

This study provides the first integrated chemical, enantioselective, and preliminary biological characterization of the leaf essential oil of *Aristolochia lingulata* Ule ex Pilg. The volatile fraction exhibited a chemically diverse profile dominated by sesquiterpene hydrocarbons and oxygenated sesquiterpenes, with 57 constituents identified and quantified across chromatographic columns of different polarity. The occurrence of compounds such as dauca-5,8-diene, *trans*-(*E*)-nerolidol, β-chamigrene, β-cyclocitral, limonene, δ-amorphene, τ-muurolol, and manool further highlights the distinctive phytochemical composition of this poorly investigated Neotropical species. Enantioselective analysis additionally contributed information on the stereochemical distribution of selected chiral metabolites, broadening the chemotaxonomic and phytochemical value of the study.

From a biological perspective, the essential oil showed moderate inhibition of acetylcholinesterase and pancreatic lipase, whereas inhibition of butyrylcholinesterase was weaker. In contrast, the DPPH and ABTS assays indicated limited radical scavenging capacity, suggesting that the biological relevance of the oil is more closely associated with enzyme modulation than with direct antioxidant effects. Overall, these findings expand current knowledge of the volatile chemistry and biological properties of *A. lingulata* and support further studies aimed at identifying the constituents responsible for the observed activities, evaluating possible synergistic interactions, clarifying mechanisms of enzyme inhibition, and establishing the toxicological profile of the oil. Given the known safety concerns associated with some members of *Aristolochia*, future pharmacological investigation should be accompanied by rigorous toxicological assessment before any potential application is considered.

## Figures and Tables

**Table 1:** Chemical analysis of *Aristolochia lingulata* Ule ex Pilg. essential oil on two stationary phases of different polarity (5%-phenyl methyl polysiloxane and polyethylene glycol).

No.	Compounds		5% Phenyl Methyl Polysiloxane				Ref.		Polyethylene Glycol				Ref.
		RT	LRI ^a^	LRI ^b^	%	σ		RT	LRI ^a^	LRI ^b^	%	σ	
1	*α*-pinene	4.03	928	932	1.1	0.04	[21]	1.87	1017	1022	1.1	0.02	[22]
2	camphene	4.42	941	946	0.7	0.02	[21]	2.31	1055	1053	0.7	0.02	[23]
3	*β*-pinene	5.21	969	974	0.4	0.01	[21]	2.86	1101	1105	0.4	0.03	[24]
4	*β*-myrcene	5.80	989	988	0.2	0.01	[21]	3.91	1157	1159	0.2	0.01	[22]
5	6-methyl-5-hepten-2-ol	5.87	991	989	0.6	0.02	[21]	9.93	1463	1468	0.6	0.04	[25]
6	Limonene	6.84	1023	1024	4.4	0.08	[21]	4.47	1186	1184	4.1	0.08	[26]
7	propyl tiglate	6.98	1027	1032	0.8	0.02	[21]	6.77	1300	-	0.8	0.02	§
8	ethanone	7.27	1037	1042	0.7	0.02	[21]	12.68	1617	1620	0.4	0.01	[27]
9	linalool	9.08	1095	1095	0.6	0.01	[21]	11.43	1546	1547	0.7	0.05	[28]
10	sorbic acid	10.10	1093	1093	4.6	0.01	[21]	8.72	1496	1490	3.9	0.22	[29]
11	*β*-thujone	10.84	1118	1112	1.8	0.03	[21]	10.59	1448	1451	1.2	0.06	[30]
12	*β*-cyclocitral	12.44	1209	1217	4.8	0.14	[21]	12.42	1602	1601	4.5	0.22	[31]
13	*cis*-geraniol	12.74	1221	1227	0.5	0.02	[21]	15.57	1796	1798	0.6	0.18	[32]
14	isobornyl acetate	14.22	1275	1283	0.3	0.01	[21]	11.78	1566	1568	0.1	0.01	[33]
15	*α*-cubebene	15.86	1337	1347	0.3	0.01	[21]	9.55	1442	1449	0.5	0.03	[34]
16	unidentified (MW = 136)	16.14	1348	-	0.2	0.01	-	9.92	1462	-	0.3	0.02	-
17	unidentified (MW = 204)	16.36	1357	-	0.4	0.03	-	9.97	1465	-	0.3	0.02	-
18	ylangene	16.48	1362	1372	0.7	0.01	[21]	10.12	1473	1472	0.9	0.06	[34]
19	*β*-cubebene	16.83	1376	1386	0.2	0.01	[21]	10.99	1521	1525	0.4	0.02	[35]
20	*β*-elemene	16.93	1380	1389	1.3	0.05	[21]	11.91	1573	1573	1.4	0.08	[34]
21	2-*epi*-*α*-funebrene	17.51	1403	1411	0.9	0.02	[21]	11.56	1554	-	0.8	0.04	§
22	*β*-funebrene	17.76	1413	1413	0.6	0.01	[21]	11.85	1570	-	0.5	0.07	§
23	unidentified (mw = 204)	17.94	1420	1434	0.1	0.01	[21]	14.23	1711	-	0.4	0.05	-
24	aromadendrene	18.14	1428	1439	0.3	0.03	[21]	12.17	1588	1589	0.2	0.01	[36]
25	cadina-1(6),4-diene	19.02	1463	1461	3.8	0.09	[21]	13.54	1670	-	3.8	0.17	§
26	dauca-5,8-diene	19.07	1465	1471	11.1	0.07	[21]	13.85	1688	-	10.9	0.15	§
27	*γ*-gurjunene	19.15	1468	1475	1.4	0.04	[21]	13.96	1696	1689	1.7	0.19	[37]
28	*β*-chamigrene	19.37	1476	1476	5.0	0.14	[21]	14.07	1701	1702	5.0	0.14	[38]
29	*α*-muurolene	19.53	1483	1483	0.7	0.02	[21]	14.13	1705	1707	0.8	0.13	[39]
30	*trans*-muurola-4(14),5-diene	19.69	1489	1493	0.5	0.01	[21]	14.92	1755	-	0.4	0.02	§
31	epizonarene	19.83	1495	1501	3.5	0.08	[21]	13.94	1669	1672	3.6	0.11	[40]
32	δ-amorphene	20.09	1509	1511	4.8	0.17	[21]	14.67	1739	-	4.6	0.07	§
33	unidentified (MW = 204)	20.36	1521	-	0.5	0.08	-	12.17	1588	-	0.2	0.01	-
34	unidentified (MW = 200)	20.47	1526	-	0.2	0.01	-	17.65	1936	-	0.2	0.01	-
35	*γ*-cuprenene	20.72	1537	1532	0.9	0.02	[21]	12.76	1623	-	0.8	0.08	§
36	*α*-calacorene	20.92	1546	1544	0.2	0.01	[21]	17.03	1892	1895	0.5	0.03	[41]
37	*trans*-(*E*)-nerolidol	21.12	1555	1561	11.3	0.13	[21]	19.10	2037	2039	11.8	0.14	[42]
38	spatulenol	21.22	1560	1568	1.4	0.03	[21]	20.10	2110	2113	1.4	0.16	[43]
39	unidentified (MW = 220)	21.57	1575	-	0.6	0.07	-	18.37	1987	-	0.5	0.17	-
40	unidentified (MW = 220)	21.88	1589	-	0.4	0.14	-	18.33	1983	-	0.2	0.02	-
41	14-hydroxy-caryophyllene,	22.01	1595	-	1.4	0.02		22.01	2254	-	0.9	0.07	§
42	unidentified (MW = 222)	22.06	1597	-	0.5	0.01	-	22.73	2308	-	0.9	0.06	-
43	10-*epi*-cubenol-12-nor-Ziza-6(13)-en-2-β-ol	22.18	1603	1598	0.4	0.01	[21]	20.85	2167	-	0.5	0.05	§
44	di-*epi*-1,10-cubenol	22.35	1610	1618	0.6	0.01	[21]	19.25	2049	2054	0.6	0.04	[44]
45	unidentified (MW = 222)	22.57	1620	-	0.5	0.01	-	20.44	2135	-	0.7	0.07	-
46	eremoligenol	22.64	1623	1629	2.1	0.04	[21]	20.72	2177	2178	1.7	0.18	[45]
47	hinesol	22.78	1630	1639	1.1	0.03	[21]	21.11	2183	2173	1.0	0.09	[46]
48	muurola-4,10(14)-dien-1β-ol	22.89	1635	1630	1.3	0.04	[21]	22.60	2299	-	0.7	0.05	§
49	τ-muurolol	22.95	1638	1640	3.7	0.10	[21]	21.52	2184	2185	3.4	0.19	[47]
50	guaia-3,9-diene	23.43	1660	1650	0.8	0.09	[21]	22.59	2298	-	0.7	0.05	§
51	guaia-3,10(14)-dien-11-ol	23.58	1667	1675	1.2	0.02	[21]	22.69	2305	-	1.0	0.07	§
52	unidentified (MW = 236)	24.53	1711	-	0.4	0.01	-	20.30	2125	-	0.8	0.10	-
53	unidentified (MW = 218)	26.01	1784	-	0.5	0.15	-	26.13	2564	-	0.7	0.10	-
54	unidentified (MW = 222)	29.24	1951	-	0.6	0.02	-	27.61	27.61	-	0.5	0.06	-
55	manool	30.91	2042	2048	4.4	0.12	[21]	25.11	2485	-	4.6	0.13	§
56	unidentified (mw = 290)	32.11	2109	-	0.7	0.01	-	28.56	2479	-	0.6	0.16	-
57	unidentified (mw = 280)	33.41	2185	-	0.5	0.01	-	31.66	2748	-	0.6	0.02	-
	monoterpene hydrocarbons				6.8						6.5		
	oxygenated monoterpenes				7.7						7.0		
	sesquiterpene hydrocarbons				36.2						36.8		
	oxygenated sesquiterpenes				25.3						23.7		
	oxygenated diterpenes				4.4						4.6		
	Others				13.1						12.7		
	Total				93.5						91.3		

^a^ Calculated linear retention index; ^b^ Linear retention index based on reference literature (Ref.); MW = molecular weight; § = identification based on MS only.

**Table 2:** Enantiomeric analysis of the components of *A. lingulata* Ule ex Pilg. essential oil.

Chiral Selector	Enantiomer	LRI ^a^	LRI ^b^	E.D. %	e.e. (%)
DAC	(1*S*,5*S*)-(–)-α-pinene	915	915	100.00	100.0
DAC	(1*S*,5R)-(+)-α-pinene	917	-		
DET	(1*R*,4*S*)-(–)-camphene	922	918	45.15	9.70
DET	(1*S*,4*R*)-(+)-camphene	938	928	54.85	
DET	(1*R*,5*R*)-(+)-β-pinene	950	-	**-**	100.0
DET	(1*S*,5*S*)-(–)-β-pinene	960	957	100.00	
DET	(*S*)-(–)-limonene	1059	1056	100.00	100.0
DET	(*R*)-(+)-limonene	1073	-	-	

LRI = linear retention index; ^a^ values from enantiomerically pure standards; ^b^ calculated LRI; E.D. = enantiomeric distribution; e.e. = enantiomeric excess; DAC = 2,3-diacetyl-6-*tert*-butyldimethylsilyl-β-cyclodextrin; DET = 2,3-diethyl-6-*tert*-butyldimethylsilyl-β-cyclodextrin.

**Table 3:** Antioxidant activity of *A. lingulata* Ule ex Pilg. essential oil.

Sample	DPPH	ABTS
	SC_50_ (µg/mL) ± SD	
*A. lingulata* Ule ex Pilg. essential oil	849.7 ± 7.4	338.0 ± 3.9
Trolox	13.5 ± 1.2	11.8 ± 1.2

SC_50_: Half-maximal scavenging concentration. Values represent the mean ± standard deviation (SD) of *n* = 3 independent experiments.

## Data Availability

The data presented in this study are available from the corresponding author upon reasonable request.

## Abbreviations

The following abbreviations are used in this manuscript:

ABTS	2,2′-Azino-bis(3-ethylbenzothiazoline-6-sulfonic acid)
AChE	Acetylcholinesterase
ATCI	Acetylthiocholine iodide
BTCI	S-Butyrylthiocholine iodide
BuChE	Butyrylcholinesterase
DAC	2,3-Diacetyl-6-tert-butyldimethylsilyl-β-cyclodextrin
DET	2,3-Diethyl-6-tert-butyldimethylsilyl-β-cyclodextrin
DMSO	Dimethyl sulfoxide
DPPH	2,2-Diphenyl-1-picrylhydrazyl
DTNB	5,5′-Dithiobis(2-nitrobenzoic acid)
EO	Essential oil
FRAP	Ferric reducing antioxidant power
GC	Gas chromatography
GC-FID	Gas chromatography with flame ionization detection
GC-MS	Gas chromatography–mass spectrometry
IC_50_	Half-maximal inhibitory concentration
LRI	Linear retention index
LRIs	Linear retention indices
MAATE	Ministerio del Ambiente, Agua y Transición Ecológica del Ecuador
MW	Molecular weight
pNPB	*p*-Nitrophenyl butyrate
RRF	Relative response factor
RSD	Relative standard deviation
RT	Retention time
SC_50_	Half-maximal scavenging concentration
SD	Standard deviation
